# Dietary patterns of pregnant women, maternal excessive body weight and gestational diabetes

**DOI:** 10.11606/S1518-8787.2019053000909

**Published:** 2019-06-25

**Authors:** Daniela Cristina Candelas Zuccolotto, Lívia Castro Crivellenti, Laércio Joel Franco, Daniela Saes Sarotelli

**Affiliations:** I Universidade de São Paulo. Faculdade de Medicina de Ribeirão Preto. Programa de Pós-Graduação em Saúde Pública. Ribeirão Preto, SP, Brasil; II Universidade de São Paulo. Faculdade de Medicina de Ribeirão Preto. Departamento de Medicina Social. Ribeirão Preto, SP, Brasil

**Keywords:** Pregnant Women, Gestational Diabetes, Feeding Behavior, Risk Factors, Prenatal Nutrition, Gestantes, Diabetes, Gestacional, Comportamento Alimentar, Fatores de Risco, Nutrição Pré-Natal

## Abstract

**OBJECTIVE:**

To investigate the relationship between the dietary patterns of pregnant women with maternal excessive body weight and gestational diabetes mellitus.

**METHODS:**

A cross-sectional study conducted with a convenience sample of 785 adult pregnant women attended by the Unified Health System of Ribeirão Preto, state of São Paulo, between 2011 and 2012. Two 24-hour dietary recalls, corrected by the *multiple source method,* were employed *.* For the classification of the body mass index and the diagnosis of gestational diabetes mellitus, the criteria by *Atalah* and the World Health Organization were used, respectively. Dietary patterns were obtained by principal component analysis using the *Varimax* rotation method. The relationship between adherence to patterns, overweight and obesity was analyzed by multinomial logistic regression models and the relationship with gestational diabetes mellitus by adjusted unconditional logistic regression models.

**RESULTS:**

We identified four dietary patterns: “traditional Brazilian”; “snacks”; “coffee” and “healthy”. Women with a higher adherence to the “Healthy” (OR = 0.52; 95%CI 0.33–0.83) and “Brazilian Traditional” patterns (OR = 0.61; 95%CI 0.38–0.96) presented a lower chance of obesity, when compared to women with lower adherence, regardless of confounding factors. After adjustment for maternal excessive body weight, there was no association between dietary patterns and gestational diabetes mellitus.

**CONCLUSIONS:**

Among the pregnant women, greater adherence to “traditional Brazilian” and “healthy” patterns was inversely associated with obesity, but no relationship was identified with gestational diabetes mellitus after adjusting for excessive body weight. Prospective studies are recommended to investigate the relationship between dietary patterns, overweight and gestational diabetes mellitus, reducing the chance of reverse causality.

## RESUMO

**OBJETIVO:**

Investigar a relação entre os padrões alimentares de gestantes com o excesso de peso materno e o diabetes mellitus gestacional.

**MÉTODOS:**

Estudo transversal conduzido em uma amostra de conveniência de 785 gestantes adultas atendidas pelo Sistema Único de Saúde de Ribeirão Preto, SP, entre 2011 e 2012. Dois inquéritos recordatórios de 24 horas, corrigidos pelo *multiple source method,* foram empregados *.* Para a classificação do índice de massa corporal e do diagnóstico do diabetes mellitus **gestacional, utilizou-se os critérios de** *Atalah* e da Organização Mundial da Saúde, respectivamente. Os padrões alimentares foram obtidos por análise de componentes principais, utilizando o método de rotação *Varimax* . A relação entre a adesão aos padrões e sobrepeso e obesidade foi analisada por modelos de regressão logística multinomial e a relação entre adesão aos padrões e diabetes mellitus gestacional, por modelos de regressão logística não condicional ajustados.

**RESULTADOS:**

Identificamos quatro padrões alimentares: “tradicional brasileiro”; “lanches”; “café” e “saudável”. Observou-se que mulheres com maior adesão ao padrão “saudável” (OR = 0,52; IC95% 0,33–0,83) e “tradicional brasileiro” (OR = 0,61; IC95% 0,38–0,96) apresentaram menor chance de obesidade que as mulheres com menor adesão, independentemente de fatores de confusão. Após ajuste pelo excesso de peso materno, não houve associação entre os padrões alimentares e o diabetes mellitus gestacional.

**CONCLUSÕES:**

Entre as gestantes, a maior adesão aos padrões “tradicional brasileiro” e “saudável” foi inversamente associada à obesidade, mas nenhuma relação foi identificada com o diabetes mellitus gestacional após ajuste pelo excesso de peso. Estudos prospectivos são recomendados para investigar a relação entre padrões alimentares e excesso de peso e diabetes mellitus gestacional, reduzindo a chance de causalidade reversa.

## INTRODUCTION

Gestational diabetes mellitus (GDM), a hyperglycemia diagnosed for the first time during pregnancy, is the most common metabolic problem during pregnancy ^1^ . Its prevalence has been growing substantially ^2^ and projections suggest this increase will go on further given the expansion in the number of cases of excessive body weight among women of reproductive age ^3^ . The GDM can trigger the development of obstetric complications and losses to maternal and child health in the short and long term ^4^ ^,^ ^5^ . Thus, the identification of modifiable risk factors related to the genesis of the disease is extremely relevant.

Certain studies indicate that both nutrient and food intake alone and adherence to dietary patterns are directly related to the health of the mother-child binomial ^3^ ^,^ ^6^ . However, the approach of eating patterns is recognized as more comprehensive, considering the synergistic and inhibitory action of the simultaneous consumption of different foods and their relationship with health outcomes ^7^ ^,^ ^8^ .

In addition to being scarce, studies that evaluated the relationship between dietary patterns during gestation and GDM show inconsistent findings due to distinct characteristic of food cultures seen among the populations analyzed^9–11^. In a study conducted in China with 3063 pregnant women, higher adherence to the “sweet and seafood” pattern was directly associated with GDM ^9^ . On the other hand, Seymor et al. ^10^ , when analyzing the dietary pattern of 909 pregnant women in Singapore, identified that adherence to the diet pattern based on seafood and pasta was inversely associated with the disease ^10^ . The divergence in the results indicates that more studies should be conducted in order to elucidate the role of maternal diet in the occurrence of GDM, especially in regions with different eating behaviors.

In view of the implications of GDM on maternal-fetal health and the small number of investigations that relate the diet of pregnant women to the occurrence of the disease, the objective of this study was to evaluate the relationship between dietary patterns and GDM. Additionally, we analyzed the relationship between dietary patterns and excessive body weight, which is a convincing risk factor for GDM.

## METHODS

### Study Design and Population

A cross-sectional study was conducted with a convenience sample of 785 pregnant women users of the Unified Health System of Ribeirão Preto, state of São Paulo, between 2011 and 2012. The women were invited to participate in the study on the occasion of their oral glucose tolerance test in five laboratories that had an agreement with the Municipal Health Department, where there was higher demand for the examination. A follow-up scheme was established by trained nutritionists every day of the week. Women who met the inclusion criteria and agreed to participate in the study were interviewed. The study design was described in detail by Barbieiri et al. ^12^ .

The inclusion criteria of the study were: gestational age after the 24th week, age ≥ 20 years and pre-gestational body mass index (BMI) ≥ 20 kg/m ^2^ . Women with diabetes, those who reported usage of glucose-modifying drugs (such as glucocorticoids) and those with chronic renal failure, AIDS or cancer, were excluded.

Between the data collection period and the analysis of the results, there was a change in the diagnostic criteria ^13^ and in the estimated prevalence of GDM ^14^ , and therefore the sample size was calculated *a posteriori* . Considering a prevalence of 20% GDM among adult women ^14^ and an acceptable margin of error of 5%, the required sample size would be 512 women. A total of 1,446 pregnant women were contacted, of which 619 were excluded due to non-compliance with the study criteria, 19 did not agree to participate, 20 did not complete the examination and three presented incomplete data, totaling 785 women. Among them, 70% were interviewed between the 24^th^ and 28^th^ gestational weeks, and 30% between the 29^th^ and 39^th^.

At the time of the interview, the pregnant women were subjected to anthropometric evaluation and answered to a structured questionnaire with socioeconomic data, obstetric and family history, lifestyle and food consumption.

### Diagnosis of Gestational Diabetes Mellitus

Fasting blood samples, one and two hours after ingestion of a 75 g glucose overload, were obtained from all study participants. Determination of glycemia was performed using the glucose oxidase test. Diagnosis of GDM was based on World Health Organization criteria of 2014 ^13^ , which takes into account pregnant women with at least one of their glycemia values altered at any stage of pregnancy: fasting glycemia between 92 and 125 mg/dl, glycemia one hour after glucose overload ≥ 180 mg/dl or glycemia two hours after glucose overload between 153 and 199 mg/dl. Pregnant women who had fasting glycemia ≥ 126 mg/dL or two hours after glucose overload ≥ 200 mg/dL were considered to have previous diabetes and were excluded from the study analyses.

### Characteristics of Pregnant Women

With use of a structured questionnaire, we obtained data on age, schooling, smoking history, family history of type 2 diabetes (DM), previous GDM, and practice of physical activities. The physical activity questionnaire used was previously developed and validated for pregnant women ^15^ . However, given the difficulty of the women in responding, for this study we consider only data related to the practice of walking for locomotion or during leisure, as well as the practice of physical exercises (in minutes per week).

Gestational age was estimated based on the date of the last menstruation (DLM). DLM and pre-gestational weight data were obtained from the record on the pregnant woman’s card. At the time of the interview, measurements of weight (in kg) and height (in m) were acquired from a digital scale (TANITA model HS302) and a portable stadiometer (SANNY model ES2040), respectively. For the classification of BMI according to the gestational week, the criteria proposed by Atalah ^16^ were adopted.

### Assessment of Food Consumption and Determination of Food Patterns

Dietary intake was assessed by two 24-hour dietary recalls (R24h) obtained by trained nutritionists using the three-steps multiple pass technique (participant reporting, detailing, and review). The first R24h was obtained at the time of the interview and the second by telephone contact after at least seven days, regardless of the day of the week or season, and before the woman receives the results of biochemical exams.

The Brazilian Table of Food Composition (TACO) ^17^ was used to estimate energy and nutrients. We analyzed the diets with the aid of the application software NutWin^®18^. In order to estimate the usual diet, we used the multiple source method ^19^ , a statistical modeling technique that considers the product between the probability of consumption and the usual intake ^19^ .

In total, 481 foods and preparations were reported in the R24h. Foods with less than 20% of consumers were excluded. Other foods and preparations were grouped into 23 groups of food (grams/day) based on the nutritional value or the logic of consumption: rice; beans; meat (red meat and chicken); fruits, vegetables; pasta; breads; butter and margarine; hard and soft cheese; cold meats (mortadella, turkey breast, ham, sausage, salami); milk and yogurt; chocolate milk and cappuccino; sugar; coffee and tea; candies ( *paçoca, pavês, pé de moleque* , popsicle, ice cream, pie, candy, *beijinho, brigadeiro* , chocolate *, cocada, doce de leite* , flan, blancmange, *maria mole* , *milkshake* , *mousse* , hazelnut cocoa spread, panettone, doughnut, *sonho* , buttery biscuit, cake); natural fruit juice; artificial juice and soda; snacks, pizzas and sandwiches (fried and baked snacks, chips, sandwich, hot dog, pizza, popcorn and instant noodles); tubers; eggs; fish; olive oil; and biscuits.

Food patterns were identified by means of principal component analysis, which allows food groups to be grouped based on the degree of correlation between them. Negative factor loadings indicate that the variable is inversely associated with the factor and positive charges, wherein the food group is directly associated with the factor. To identify the number of patterns to be retained, we use eigenvalues > 1.5. After orthogonal Varimax rotation, we kept the food groups with a factorial load ≥ 0.3. In total, 18 food groups were retained within the food patterns. The tubers, eggs, fish, olive oil and biscuits groups presented low commonality and were excluded.

### Statistical Analysis

Descriptive and comparative analyses of the study variables according to dietary pattern were performed using the chi-square test for categorical variables, and ANOVA and Kruskal-Wallis for continuous variables.

Adjusted multinomial logistic regression models were used to evaluate the association between adherence to dietary patterns (in tertile) with overweight and obesity, adopting the eutrophic women as reference. For such analyses, 31 women with low weight for their gestational week were excluded. We considered the following adjustment variables for the models: age (years), gestational week at the time of interview, education (years of study), smoking (never smoked, interrupted during gestation or currently smokes), physical activity practice (minutes/week walking or exercise) and number of children.

Adjusted logistic regression models were used to assess the association between adherence to dietary patterns and GDM. GDM (yes or no) was considered a dependent variable, and dietary patterns (in tertile) were considered independent variables. Model 1 was adjusted for: age (years), gestational week at the time of the interview, previous GDM (yes; no), schooling (years of study), family history of DM (yes; no), smoking (never smoked, interrupted during gestation, or currently smokes), physical activity (minutes/week of walking or exercise) and number of children. In Model 2, in addition to the variables included in Model 1, further adjustment for maternal excessive body weight (yes or no) was considered.

The selection of adjustment variables for the models was based on the theoretical reference of food consumption influence on the occurrence of maternal excessive body weight, as well as on GDM. However, variables considered the causal pathway between exposure and outcomes, such as dietary energy, were not taken into account. A value of p < 0.05 was adopted as level of significance. The statistical analyses were performed with the aid of the application software IBM SPSS Statistics (version 24.0, SPSS Inc. Woking, Surrey, UK).

This study was approved by the Research Ethics Committee of the School and Health Center of the Ribeirão Preto Medical School, University of São Paulo (CEP/CSE-FMRP-USP-023/2015) and its execution authorized by the Municipal Secretary of Health of Ribeirão Preto (2030/10 GS).

## RESULTS

Among the 785 pregnant women investigated, 139 (17.7%) had gestational diabetes, 261 (33.2%) were classified as overweight and 187 (23.8%) as obese.

Four dietary patterns were identified: “traditional Brazilian”, “snacks”, “coffee” and “healthy”, which together accounted for about 41% of the variance, and the observed KMO was 0.56. The “traditional Brazilian” pattern was characterized by consumption of rice; bean; meat; vegetables, and inversely associated with the consumption of hard and soft cheese; snacks, pizzas and sandwiches. The “snacks” patterns was characterized by the consumption of breads; butter and margarine; milk and yogurt; hard and soft cheese; sweets; chocolate milk and *cappuccino* . The “coffee” pattern was characterized by consumption of coffee; sugar, butter and margarine. The “healthy” pattern was characterized by the consumption of vegetables; fruit and natural fruit juice; and it was inversely associated with the consumption of soda and artificial juice ( Table 1 ).

**Table 1:** Factor loading of the dietary patterns of pregnant women obtained by principal component analysis. Ribeirão Preto, state of São Paulo, 2011–2012. (n = 785)

Food group (g/day)	Feeding pattern			

	Traditional Brazilian	Snacks	Coffee	Healthy
Rice	**0.811**	-0.014	0.015	0.015
Beans	**0.738**	-0.042	-0.024	0.125
Meats (red meat and chicken)	**0.448**	0.091	-0.047	-0.165
Snacks, pizzas and sandwiches	**-0.331**	0.101	-0.232	-0.012
Pasta	-0.291	0.155	0.118	-0.290
Breads	0.159	**0.718**	0.299	-0.049
Butter and margarine	0.222	**0.547**	**0.321**	-0.145
Cold meats	0.221	**0.467**	0.026	0.037
Milk and yogurt	0.134	**0.448**	**-0.320**	0.249
Hard and soft cheese	**-0.315**	**0.440**	-0.168	0.221
Candies	-0.141	**0.330**	-0.067	-0.180
Coffee and tea	0.051	0.067	**0.787**	-0.028
Sugar	-0.009	0.134	**0.651**	0.114
Chocolate milk and cappuccino	0.101	**0.467**	**-0.520**	-0.080
Natural fruit juice	-0.185	0.083	-0.070	**0.616**
Vegetables	0.383	0.048	-0.046	**0.576**
Soda and artificial juice	0.114	0.261	-0.208	**-0.547**
Fruits	0.021	0.022	0.074	**0.544**

Variance %	11.4	10.5	9.8	9.0
Cumulative variance %	11.4	22.0	31.8	40.8

^a^ The foods whose factor loading is higher than 0.30 were kept in the matrix. Extraction method: principal component analysis. Rotation Method: *Varimax* with Kaiser normalization.

Factor loading values ≥ 0.3 or ≤ -0.3 are shown in bold.

Women with greater adherence to the “traditional Brazilian” pattern reported lower schooling, more time spent in physical activities, and lower pre-gestational BMI than women with greater adherence. There was also a difference in smoking frequency and family history of type 2 diabetes. The pregnant women with greater adherence to the “snacks” pattern were younger and reported more schooling and less time practicing physical activities than pregnant women with lower adherence. Those with higher adherence to the “coffee” pattern presented higher average age, a higher number of children and lower schooling than those with lower adherence. And women with greater adherence to the “healthy” pattern had a higher average age and reported more time spent on physical activities than women with lower adherence ( Table 2 ). However, some of these differences are not considered clinically relevant.

**Table 2:** Sociodemographic characteristics, anthropometric data and lifestyle according to dietary patterns during gestation. Ribeirão Preto, state of São Paulo, 2011–2012. (n = 785)

Mother characteristics	All	Pattern traditional Brazilian		Pattern snack		Pattern coffee		Pattern healthy	
			
		1^st^ tercile	3^rd^ tercile	1^st^ tercile	3^rd^ tercile	1^st^ tercile	3^rd^ tercile	1^st^ tercile	3^rd^ tercile
With GDM/normoglycemic	139/646	58/203	40/222	48/213	43/219	43/218	48/214	45/216	50/212

	n (%)								

Previous GDM	34 (4.3)	9 (3.4)	17 (6.5)	10 (3.8)	8 (3.1)	7 (2.7)	17 (6.5)	8 (3.1)	17 (6.5)
DM family history	205 (26.1)	67 (25.7)	82 (31.3)^a^	77 (29.5)	70 (26.7)	74 (28.4)	62 (23.7)	67 (25.7)	74 (28.2)
Smoking during pregnancy	71 (9.0)	16 (6.1)	33 (12.6)	26 (10.0)	24 (9.2)	20 (7.7)	29 (11.1)	26 (10.0)	18 (6.9)
BMI category according to gestational age									
Low weight	27 (3.4)	6 (2.3)	12 (4.6)	11 (4.2)	10 (3.8)	11 (4.2)	6 (2.3)	12 (4.6)	10 (3.8)
Eutrophia	310 (39.5)	97 (37.2)	107 (40.8)	105 (40.2)	102 (38.9)	107 (41.0)	104 (39.7)	98 (37.5)	114 (43.5)
Overweight	261 (33.2)	84 (32.2)	92 (35.1)	74 (28.4)	94 (35.9)	88 (33.7)	84 (32.1)	79 (30.3)	87 (33.2)
Obesity	187 (23.8)	74 (28.4)	51 (19.5)	71 (27.2)	56 (21.4)	55 (21.1)	68 (26.0)	72 (27.6)	51 (19.5)

	Mean (SD)								

Age (years)	27.6 (5.4)	28.1 (5.7)	27.0 (5.2)	28.5 (5.5)	26.8 (5.3)^b^	26.4 (4.8)	28.5 (5.8)^b^	26.7 (5.1)	28.6 (5.8)^b^
Schooling (years of study)	9.2 (2.7)	9.6 (2.6)	8.9 (2.7)^b^	8.7 (3.0)	9.6 (2.5)^b^	9.5 (2.3)	8.9 (2.9)^b^	9.0 (2.5)	9.3 (2.8)
Pre-gestational BMI (kg/m ^2^ )	25.9 (5.0)	26.5 (5.2)	25.3 (4.6)^b^	26.4 (5.5)	25.5 (5.0)	25.5 (4.8)	26.0 (5.0)	26.1 (5.2)	25.3 (4.9)
Number of children	1.2 (1.2)	1.2 (1.2)	1.1 (1.2)	1.3 (1.3)	1.1 (1.2)	0.9 (1.1)	1.5 (1.4)^b^	1.2 (1.3)	1.2 (1.2)

	Median (P25; P75)								

Physical activity^d^	40 (0; 140)	35 (0; 120)	60 (0; 150)^c^	50 (0; 150)	40 (0; 120)^c^	40 (0; 122)	50 (0; 140)	40 (0; 120)	60 (0; 150)^c^

GDM: gestational diabetes mellitus; DM: diabetes mellitus; BMI: body mass index

^a^ p < 0.05, according to the X ^2^ test.

^b^ p < 0.05, according to ANOVA.

^c^ p < 0.05, according to the Kruskal-Wallis test.

^d^ Physical activity (minutes per week of walking or exercise).

According to multinomial logistic regression models adjusted for age, gestational week at the time of the interview, schooling, smoking, number of children and physical activity, the pregnant women with greater adherence to the “traditional Brazilian” and “healthy” patterns showed less chance of obesity than those with lower adherence. Pregnant women classified as within the second tertile of adherence to the “snacks” pattern were more likely to be overweight than those in the first tertile, but this relationship was observed only at the intermediate level of adherence to the pattern ( Table 3 ).

**Table 3:** Association between dietary patterns during pregnancy with overweight and obesity. Ribeirão Preto, state of São Paulo, 2011–2012a. (n = 754)

Variable	1st tercile	2nd tercile		3rd tercile	
	
		OR	95%CI	OR	95%CI
Traditional Brazilian pattern					

Overweight					
Adjusted model^b^	1.00	0.85	0.56–1.29	1.06	0.70–1.60
Obesity					
Adjusted model^b^	1.00	0.70	0.45–1.08	0.61	0.38–0.96

Snacks pattern					

Overweight					
Adjusted model^b^	1.00	1.55	1.01–2.36	1.40	0.92–2.15
Obesity					
Adjusted model^b^	1.00	0.88	0.56–1.38	0.86	0.55–1.35

Coffee pattern					

Overweight					
Adjusted model^b^	1.00	1.25	0.83–1.89	1.13	0.75–1.72
Obesity					
Adjusted model^b^	1.00	1.27	0.81–2.00	1.12	0.71–1.76

Healthy pattern					

Overweight					
Adjusted model^b^	1.00	1.13	0.74–1.71	0.84	0.55–1.29
Obesity					
Adjusted model^b^	1.00	0.89	0.57–1.38	0.52	0.33–0.83

^a^ Multinomial logistic regression models, considering eutrophic models as reference.

^b^ Multinomial logistic regression models adjusted for age, gestational week at the time of interview, schooling (years of study), smoking (never smoked, interrupted during gestation, and currently smokes), physical activity practice (minutes/week of walking for locomotion or physical exercise) and number of children.

In non-conditional logistic regression models adjusted for age, gestational week at the time of the interview, schooling, previous GDM, family history of DM, smoking, number of children and physical activity, it was found that women with higher adherence to the “traditional Brazilian” had a lower chance of GDM. However, this association was not independent of maternal excessive body weight ( Table 4 ).

**Table 4:** Relationship between dietary patterns during gestation and gestational diabetes mellitus. Ribeirão Preto, state of São Paulo, 2011–2012a. (n = 785)

Variable	1^st^ tercile	2^nd^ tercile	3^rd^ tercile	p trend
	
		OR (95%CI)	OR (95%CI)	
Traditional Brazilian pattern				
Model 1^b^	1.00	0.64 (0.41–1.02)	0.62 (0.39–0.99)	0.04
Model 2^c^	1.00	0.66 (0.41–1.05)	0.64 (0.39–1.04)	0.06
Snacks pattern				
Model 1^b^	1.00	1.06 (0.67–1.69)	1.01 (0.63–1.63)	0.95
Model 2^c^	1.00	1.00 (0.63–1.61)	0.96 (0.59–1.55)	0.88
Coffee pattern				
Model 1^b^	1.00	1.01 (0.63–1.63)	0.97 (0.60–1.58)	0.91
Model 2^c^	1.00	1.00 (0.63–1.63)	0.97 (0.59–1.59)	0.92
Healthy pattern				
Model 1^b^	1.00	0.92 (0.57–1.48)	0.97 (0.61–1.56)	0.91
Model 2^c^	1.00	0.91 (0.57–1.47)	1.04 (0.64–1.68)	0.87

^a^ Non-conditional logistic regression models, considering normoglycemic women as reference.

^b^ Model 1 was adjusted for: age (years), gestational week at the time of the interview, previous GDM (yes/no), schooling (years of study), family history of DM (yes/no), smoking (never smoked, interrupted during gestation, or currently smokes), physical activity (minutes/week of walking or exercise) and number of children.

^c^ Model 2: Additional adjustment for maternal excessive body weight (yes/no).

## DISCUSSION

In this study, pregnant women with greater adherence to the “healthy” and “traditional Brazilian” patterns presented a lower chance of obesity. On the other hand, women classified in the intermediate level of adherence to the “snacks” pattern presented a greater chance of being overweight. Regarding GDM, the findings suggest that maternal excessive body weight portrays relevant causality with respect to the inverse association between the “traditional Brazilian” dietary pattern and GDM.

The prevalence of obesity has been increasing exponentially, which can be partially attributed to changes in the population’s eating behavior, where meal-based dietary patterns are replaced by those based on ready-to-eat industrialized products ^20^ . Our findings support this hypothesis, since women with greater adherence to the “traditional Brazilian” pattern, composed of typical food markers for meals, such as rice, beans, meats, vegetables, characterized by a lower consumption of snacks, pizzas and sandwiches, presented a lower chance of obesity.

The inverse association between adherence to the “healthy” pattern and obesity in pregnant women corroborates the scientific evidence of a possible protective effect of food intake with adequate nutrient intake and low caloric density in the energy balance. Fruits, vegetables are also high in fiber, which have the capacity to reduce gastric emptying time and promote satiety ^21^ . Moreover, the “healthy” pattern was inversely associated with the consumption of soda and artificial juices, which are considered relevant risk factors for obesity due to their high sugar content, high glycemic index and, consequently, low satiety capability. Sugary drinks are rapidly absorbed by the body and do not stimulate the signs of satiety in the same way as solid foods. Moreover, they are ineffective in stimulating insulin production, one of the physiological signals for energy balance ^22^ . In addition, women with greater adherence to “traditional Brazilian” and “healthy” patterns reported longer walking and exercise time, which could contribute to a greater energy expenditure and adequate weight maintenance ^23^ ; however, the associations were independent of physical activity practice.

The positive association found between the “snack” pattern and overweight is consistent with the literature ^24^ ^,^ ^25^ . However, in this study, this association was significant only for the second tertile, compared to the first. The “snacks” pattern consisted of breads, butter and margarine, cold meats, hard and soft cheese, and sweets, which suggests a pattern of food substitution from main meals to snacks. Frequent consumption of these foods with high energy density can lead to damage in the neuronal circuits involved with the regulation of appetite and satiety ^26^ .

Obesity is considered a significant risk factor for GDM. The accumulation of body fat, associated with physiological changes mediated by placental hormones in pregnancy, can increase insulin resistance and predisposition to GDM. It is known that DMG is due to both exacerbated insulin resistance and previous dysfunction of β-cells ^27^ . In this study, adjustment for maternal excessive body weight attenuated the association between the “traditional Brazilian” pattern and GDM.

Studies that investigated the relationship between dietary patterns during pregnancy and GDM are scarce and were conducted in regions with different eating habits to Brazilian culture, limiting the comparison of the findings ^3^ ^,^ ^6^ ^,^ ^9^ ^,^ ^10^ ^,^ ^19^ ^,^ ^28^ . A cohort study conducted in Singapore among 909 pregnant women showed that women with higher adherence to a pattern rich in vegetables, fruits, white rice, meats and fish with low consumption of potato chips, hamburgers and sugary drinks had a lower chance of GDM ^10^ . This pattern consists of some foods found in the “traditional Brazilian” and “healthy” standards of this study; however, these patterns were not related to the outcome.

Among the results of the study, a higher proportion of women with GDM were classified as within the highest tertile of the “healthy” pattern, which was not expected and cannot be explained in the literature. One of the hypotheses of the present study was an inverse association between adherence to the “healthy” pattern and GDM, which was not confirmed. According to the literature, the results are controversial ^6^ ^,^ ^9^ ^,^ ^10^ ^,^ ^29^ . It is suggested that dietary patterns rich in fruits and vegetables have a protective effect on the development of diseases such as GDM, given the low energy density, glycemic load and the high antioxidant and phytochemical content of these foods ^21^ . However, dietary habits are specific to each population, with different ethnicities and genetic predispositions, which could partially explain the disagreement between studies. Exposure to modifiable risk factors during the pre-gestational period, which was not considered in the present study, may also have influenced the results found.

A possible justification for the lack of association between the “healthy” and “snack” patterns and the outcomes analyzed is the fact that these food consumption patterns are more subject to social desirability bias. One study showed that individuals with higher social desirability tend to report higher consumption of fruits, vegetables, and lower food intake of breads and biscuits, thus attenuating associations ^30^ .

One cannot ignore the hypothesis that a voluntary change in food consumption may occur during pregnancy in order to promote the growth and development of the baby. However, the evidence regarding dietary changes in this life cycle is controversial ^31^ .

Evaluating dietary intake through analysis of dietary patterns is considered an adequate strategy, since it contemplates the diet as a whole, supplying the limitation of approaches that evaluate nutrients and food items alone ^7^ ^,^ ^8^ . Among the strengths of the study, we highlight the use of the *multiple source method* ^19^ for the estimation of the usual diet, and the use of the World Health Organization criteria from 2014 ^13^ for the diagnosis of GDM, which were accepted by the International Federation of Gynecology and Obstetrics ^32^ . The main limitation of the study is its cross-sectional design, which made it impossible to identify a temporal relationship between dietary patterns and the outcomes investigated. Reverse causality may also have occurred, since 46% of the pregnant women evaluated had excessive body weight during the pre-gestational period. However, neither the pregnant women nor the interviewers knew the results of the diagnostic test for GDM at the time of the interview, which reduced the chance of information bias in the investigation of this outcome. Data on weight gain during the first trimester of pregnancy were not collected, which made it impossible to estimate the adequacy of weight gain; thus, the models were adjusted for excessive body weight according to gestational age as an indicator of both pre-gestational excessive body weight and excessive weight gain. Social desirability was not investigated in this study, making it impossible to confirm the hypothesis of reporting bias. Another limitation inherent to studies evaluating dietary data is a possible underreporting of dietary intake. In addition, the use of logistic regression in cross-sectional studies whose outcome is highly prevalent has been criticized by some authors. However, this measure of association was used because it is recommended for cross-sectional studies investigating the effect of an exploratory variable on the occurrence of a health outcome ^33^ . The data presented here suggest a lower chance of obesity among pregnant women with greater adherence to “traditional Brazilian” and “healthy” patterns. There was no association between dietary patterns and GDM regardless of maternal excessive body weight. Prospective studies are recommended to investigate the relationship between dietary patterns and excessive body weight and GDM, reducing the chance of reverse causality.
