# Erratum

**DOI:** 10.11606/s1518-8787.2019053000909err

**Published:** 2019-12-16

In **“Dietary patterns of pregnant women, maternal excessive body weight and gestational diabetes”**. Rev. Saude Publica [online]. 2019, vol.53:52, ISSN 1518-8787, http://dx.doi.org/10.11606/s1518-8787.2019053000909, the RSP corrects the author’s last name.

**Where you read:**

Daniela Saes Sarotelli

**You should read:**

Daniela Saes Sartorelli

