# Supplementary material for: Erratum
Source: Rev Saude Publica. 2019 Dec 16;53:52err. doi: 10.11606/s1518-8787.2019053000909err (PMC6919279; doi:10.11606/s1518-8787.2019053000909err)
Supplement: Translation

# Translation: Errata

No artigo **“Padrões alimentares de gestantes, excesso de peso materno e diabetes gestacional”**. Rev. Saude Publica [online]. 2019, vol.53:52, ISSN 1518-8787, http://dx.doi.org/10.11606/s1518-8787.2019053000909, a RSP corrige o sobrenome do autor.

**Onde se lê:**

Daniela Saes Sarotelli

**Leia-se:**

Daniela Saes Sartorelli
